# Potential vectors of Leishmaniases in the Environmental Protection Area and Tinguá Federal Biological Reserve, Municipality of Nova Iguaçu, Rio de Janeiro, Brazil

**DOI:** 10.1111/mve.70031

**Published:** 2025-11-17

**Authors:** Antônio L. F. Santana, Alfredo C. R. Azevedo, Margarete M. S. Afonso, Bruno M. Carvalho, Vanessa R. Vieira, Simone M. Costa, Júlia S. Silva, Thais A. Pereira, Daniela P. Pereira, Maurício L. Vilela

**Affiliations:** ^1^ Laboratório Interdisciplinar de Vigilância Entomológica em Diptera e Hemiptera ‐LIVEDIH/Instituto Oswaldo Cruz/ FIOCRUZ Rio de Janeiro Brazil; ^2^ Barcelona Supercomputing Center/Centro Nacional de Supercomputación Barcelona Spain; ^3^ Laboratório de Diptera/Inatituto Oswaldo Cruz/FIOCRUZ Rio de Janeiro Brazil; ^4^ Laboratório de Biologia Molecular e Doenças Endêmicas/Instituto Oswaldo Cruz/FIOCRUZ Rio de Janeiro Brazil

**Keywords:** Leishmaniases, Rio de Janeiro, standardized index of species abundance, Tinguá Federal Biological Reserve, vectors

## Abstract

Leishmaniases are zoonotic diseases with outbreaks influenced by environmental factors that can alter their epidemiological profiles. They are transmitted to humans and other vertebrates through the bite of a female phlebotomine infected with parasites of the *Leishmania* genus. The aim of this study was to conduct an entomological survey of the phlebotomine fauna to determine the distribution of potential vectors of leishmaniases, as well as to diagnose *Leishmania* spp. and evaluate food content. A sample study on potential leishmaniases vectors was conducted in an Environmental Protection Area (EPA) and Tinguá Federal Biological Reserve (TFBR), Nova Iguaçu municipality, in Rio de Janeiro State. Light traps were used to collect insects in the two study areas from September 2019 to March 2020. The data were obtained from six monitoring stations (MSs): MS_1_, MS_2_ and MS_3_ (EPA), and MS_4_, MS_5_ and MS_6_ (TFBR). Traps were installed in intradomicile, peridomicile, and residual forests in the EPA, while they were set up in wild animal burrows and rock formations in the TFBR. Phlebotomine samples (Diptera, Psychodidae) obtained from different MS'_s_ were used to estimate the standardized index of species abundance (SISA), diagnose specimens as *Leishmania* spp., and analyse the blood food content of the female sand flies. Seven primary or potential vectors were detected in relation to the total number of collected sand flies. These included *Nyssomyia intermedia* in the intradomicile, peridomicile, and residual forests of the EPA. This species was not detected in the TFBR, but other potential vectors were observed in both areas. During the diagnosis of *Leishmania* spp. in the sand flies, one specimen of *Psychodopygus hirsutus hirsutus* was positive for *Leishmania* (*Viannia*) *braziliensis* in the peridomicile (MS_3_). A specimen of *Psychodopygus davisi* was observed feeding on *Tamandua tetradactyla* in MS_5_ (TFBR), a potential reservoir of *Leishmania*. The presence of primary vectors, potential vectors, *L*. (*V*.) *braziliensis*, and a natural reservoir indicated the possible existence of a sylvatic and domestic transmission cycle in the American tegumentary leishmaniasis region.

## Graphical abstract

- This study was to describe the composition of sand fly fauna, detect potential vectors of *Leishmania* spp. in the Tinguá district, an ATL endemic, Rio de Janeiro, Brazil.
- Seven species of vectorial importance in the epidemiological cycle of leishmaniasis was observed: *Nyssomyia intermedia*, *Migonemyia migonei*, *Pintomyia fischeri*, *Psychodopygus hirsutus hirsutus*, *Ps*. *ayrozai*, *Ps*. *davisi* and *Evandromyia edwardsi*.
- The presence of primary/potential vectors, *L*. (*V*.) *braziliensis,* natural reservoir in Tinguá, drawing attention to the probability of a transmission cycle that could be both wild and domestic for ATL.

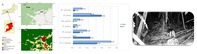

## INTRODUCTION

Leishmaniases are characterized by a group of diseases defined as zoonoses caused by digenetically flagellated protozoa of the order Kinetoplastida and family Trypanosomatidae, whose etiological agents are parasites of the genus *Leishmania* Ross. They are widely distributed and occur in 98 countries in Central America, South America, Africa, India, East and Central Asia, and on the shores of the Mediterranean (Alvar et al., 2012; Ruiz‐Postigo et al., 2021; WHO, 2016). Due to the clinical complexity of the disease and the diversity of *Leishmania* species, vectors, and domestic or wild reservoirs, different transmission cycles can occur (Desjeux, 2004; Maia‐Elkhoury et al., 2018; OPS, 2023). The distribution of Leishmaniases is associated with poverty and social, economic, and environmental conditions that directly influence its epidemiology (Alvar et al., 2006; Franke et al., 2002; Maia‐Elkhoury et al., 2016). As a result, Leishmaniasis can be observed in urban areas of several large cities in Brazil. The Metropolitan Region of Nova Iguaçu, State of Rio de Janeiro, Brazil, where socio‐environmental degradation is linked to a long history of the inability to manage natural and social assets, leading to outbreaks of viral and parasitic diseases, can be included in this context. Outbreaks, in addition to behavioural issues, are also linked to a lack of public safety, sanitation, and educational policies (Prefeitura Municipal de Nova Iguaçu, 2021).

Over the past 30 years, several outbreaks of American tegumentary leishmaniasis (ATL) have been recorded in Brazilian municipalities. The presence of *Nyssomyia intermedia* has been highlighted in studies of potential vectors in the state of Rio de Janeiro, suggesting that it is the main vector of *Leishmania* (*Viannia*) *braziliensis* (Aguiar et al., 2014; Azevedo et al., 2015; Gouveia et al., 2012; Meneses et al., 2005; Pita‐Pereira et al., 2005; Rangel et al., 2018). However, other species, such as *Migonemyia migonei* (Pita‐Pereira et al., 2005; Rangel et al., 2018; Rangel & Lainson, 2009), *Pintomyia fischeri* (Aguiar et al., 2014; Pita‐Pereira et al., 2011; Rangel et al., 2018), *Evandromyia edwardsi* (Capucci et al., 2023; Serra e Meira et al., 2022) and *Psychodopygus davisi* (de Souza et al., 2016; Gil et al., 2003; Rangel et al., 2018), found in the State of Rio de Janeiro, have been mentioned as potential vectors for *L*. (*V*.) *braziliensis* and the latter, together with *Ps*. *hirsutus hirsutus* (Gil et al., 2003) and *Ps*. *ayrozai* (Rangel et al., 2018; Rangel & Lainson, 2009; Vilela et al., 2013) to *L*. (*V*.) *naiffi*. From 2007 to 2024, 1164 cases of ATL were reported in the State of Rio de Janeiro, of which the municipality of Nova Iguaçu was responsible for 1% of the records, being the tenth largest in number of cases. For AVL, 142 cases were recorded in the state in the same period, with Nova Iguaçu reporting one case (SINAN, 2025). The aim of this study was to describe the composition of sand fly fauna and detect potential vectors of *Leishmania* spp. in the Tinguá district, an ATL‐endemic area in the State of Rio de Janeiro, southeast Brazil.

## MATERIALS AND METHODS

### Study area

The study was carried out in the Tinguá District, located in the far north of the municipality of Nova Iguaçu, in the State of Rio de Janeiro. More than 27% of the territory is represented by Conservation Unit areas, characterized as the Atlantic Forest biome (Prefeitura de Nova Iguaçu, 2022).

**FIGURE 1:**
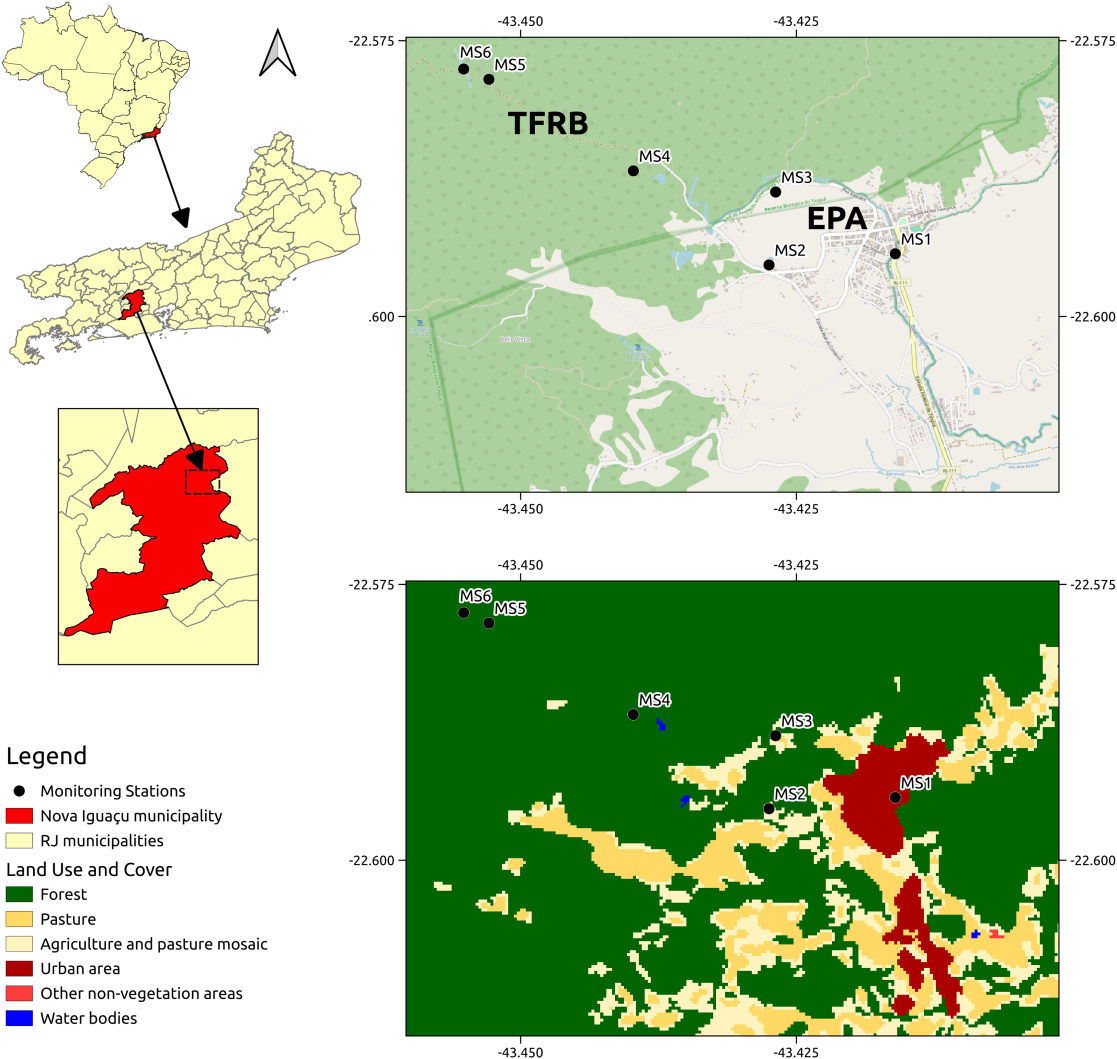
Study area, with Monitoring Stations (MSs) in the Tinguá Environmental Protection Area (EPA) and the Tinguá Biological Reserve (TFBR), Nova Iguaçu Municipality, Rio de Janeiro State, Brazil (*Source*: http://www.cmni.rj.gov.br/wp/nova‐iguacu/mapa‐da‐cidadE).

The Environmental Protection Area (EPA) has rural and peri‐urban characteristics, as well as an important tourist flow due to its landscape, which forms a contiguous territory to the Tinguá Federal Biological Reserve (TFBR) and covers an area of approximately 5400 ha in the northern region of the municipality (Figure 1).

The TFBR is in the Serra do Mar Biodiversity Corridor, in the Atlantic Forest biome in the State of Rio de Janeiro, approximately 60 km from the capital. It is located between latitudes 22°22′20″S and 22°45′00″S, and longitudes 43°40′00″W and 43°05′40″W. It has 26,260 hectares distributed among the municipalities of Nova Iguaçu (56%), Duque de Caxias (37%), Petrópolis (4%), and Miguel Pereira (3%).

This reserve is extremely important for the conservation of fauna, flora, and abiotic resources. The forest formations are Submontane Dense Ombrophilous, Montane Dense Ombrophilous and Altomontane Dense Ombrophilous. The climate in the region is tropical highland according to the Köppen classification, characterized by an average temperature of the coldest month that is always above 18°C and a short dry season that is offset by high rainfall totals (IBAMA, 2006) (Figure 1).

The sand flies species were collected at the EPA and inside the TFBR between September 2019 and March 2020. The monitoring stations (MSs) were chosen according to the criteria established by the Ministry of Health (2017) (Table 1).

**TABLE 1:** Locations of collects, according to Monitoring Stations (MSs), in Tinguá, in the Municipality of Nova Iguaçu, State of Rio de Janeiro, Brazil.

Collect site	Monitoring station	Environment	Geographic coordinates	Features of the collect site
Periurban Area	MS_1_	Peridomicile	S: 22° 35,578 W: 43° 25,107	Tinguá Center
(EPA)		Peridomicile	S: 22° 35,652 W: 43° 24,968	Area with several
		Peridomicile	S: 22° 35,627 W: 43° 25,106	Residences with
		Peridomicile	S: 22° 35,505 W: 43° 24,777	Shelters (corral,
		Peridomicile	S: 22° 35,508 W: 43° 24,791	chicken coop and
		Peridomicile	S: 22° 35,278 W: 43° 25,310	kennel)
Rural Area	MS_2_	Intradomicile	S: 22° 55,732 W: 43° 25,666	Residence with
(EPA)		Peridomicile	S: 22° 35,720 W: 43° 25,659	chicken coop and kennel.
		Forest	S: 22° 35,645 W: 43° 25,650	Area with fruit trees, wild and synanthropic animals.
Rural Area	MS_3_	Intradomicile	S: 22° 35,299 W: 43° 25,617	Residence with chicken coop and other domestic animals
(EPA)		Peridomicile	S: 22° 35,323 W: 43° 25,613	(dogs, cats and horses) and with a lot of organic matter in the soil.
		Forest	S: 22° 35,336 W: 43° 25,517	Area with fruit trees, wild and synanthropic animals.
Forest Area	MS_4_	Forest	S: 22° 35,208 W: 43° 26,387	Forest area with bamboo groves and wild animals.
(TFBR)	MS_5_	Forest	S: 22° 34,710 W: 43° 17,173	Forest area, with hollow trees and wild animal den
	MS_6_	Forest	S: 22° 34,654 W: 43° 27,310	Forest area, close to a rocky slope and wild animal den.

### Phlebotomine collected

HP light traps were used (Pugedo et al., 2005) and the original nylon cages were adapted with plastic pots (Fuzari‐Rodrigues et al., 2013) containing 250 mL of 80% alcohol. Traps were installed between 3 and 4 p.m. on the first day and removed between 9 and 10 a.m. on the fourth day. Traps were installed at different collect sites and MSs. Seven traps were set in the EPA: one in MS_1_ in the peridomicile (backyard), three in MS_2_ and three in MS_3_ in the intradomicile (balcony), peridomicile (chicken coop or corral), and residual forest areas. Three traps were set up in the TFBR, i.e., one in each MS. These were set up inside the reserve in rock crevices, holes in trees and animal burrows.

### Phlebotomine processing and taxonomic identification

After separating the sand flies from the other insects, they were subjected to clarification and diaphanization (Vilela et al., 2018). Species names were abbreviated according to Marcondes (2007) for taxonomic classification, and species were identified by observing morphological characteristics using the dichotomous key proposed by Galati (2024).

### *Detection of* Leishmania *spp. DNA*

Female (not engorged) sand flies were identified taxonomically (the head and the last three abdominal segments were sectioned for mounting and specific identification with the rest of the body sent for molecular analysis), separated individually into Eppendorf tubes labelled according to the collect area, species and sex, and sent to the laboratory for molecular assays. Males were used as controls for all stages of the diagnostic test, and females were analysed individually for the parasite according to Lins et al. (2002) and Pita‐Pereira et al. (2005). The samples were processed in the lysis buffer (10 mM Tris–HCl pH 9.2 containing 10 mM EDTA and 100 μg/mL proteinase K) and stored (−20°C) until the total DNA was extracted. DNA was extracted from the lysates using the Wizard SV Genomic DNA Purification System (PROMEGA) according to the manufacturer's specifications. All stages of DNA extraction were monitored by including negative control samples (male insects collected in the field), and all materials used in this stage were properly decontaminated with chlorine and UV exposure.

For the Hot‐start PCR multiplex reaction, two pairs of primers that simultaneously amplify a 120 bp product, referring to *Leishmania* kDNA (in the case of positive female samples), and a 220 bp product, corresponding to sand fly DNA (in the case of all samples containing males and females), were used. The first pair amplifies the conserved kDNA minicircle region: primer A [5′ GGC CCA CTA TAT TAC ACC AAC CCC 3′] and primer B [5′ GGG GTA GGG GCG TTC TGC GAA 3′] (Passos et al., 1996); the second pair amplifies a phlebotomine‐specific constitutive gene (cacophony): 5Llcac [5′ GTG GCC GAA CAT AAT GTT AG 3′] and 3Llcac [5′ CCA CGA ACA AGT TCA ACA TC 3′] (Lins et al., 2002). All amplified products were visualized using 2% agarose gel electrophoresis and stained using Nancy‐520.

The amplified PCR product was also analysed using solid‐phase hybridization and the Dot‐Blot technique. A subgenus‐specific or species‐specific probe, marked with biotin at the 5' end, revealed using a chemiluminescence solution was used.

### Food source detection

For the analysis of food content, female (engorged) collected and taxonomic identification (the head and the last three abdominal segments were sectioned for mounting and specific identification with the rest of the body sent for molecular analysis) were individually separated in Eppendorf tubes labelled according to the collect area, species and sex, and sent to the laboratory for molecular testing. Primers that amplify the cytochrome b gene (cyt b) were used: 3′ CCC CTC AGA ATG ATA TTT GTC CTC A 5′ and 3′ CCA TCC AAC ATC TCA GCA TGA GA AA 5′ (Peña et al., 2012) and the products obtained for this gene were purified using the Wizard SV PCR Clean‐up System kit (PROMEGA) and sequenced with the same primers used for PCR. Sequencing was carried out on an automatic sequencer (ABI PRISM BigDye Terminator Cycle Sequencing) at the Oswaldo Cruz Foundation (IOC) (Genomic Platform ‐ DNA sequencing, PDTIS‐FIOCRUZ). The obtained sequences were aligned and compared with those deposited in the NCBI nucleotide database [http://blast.ncbi.nlm.nih.gov/Blast].

### Analysis of collected sand fly species caught in the EPA and TFBR

The abundance of species at the MSs was estimated using the standardized index of species abundance (SISA) (Roberts & His, 1979). The analysis considered samples from six MSs: intradomicile (MS_2_ + MS_3_), peridomicile (MS_1_ + MS_2_ + MS_3_), and residual forest (MS_2_ + MS_3_) in the EPA, and TFBR (MS_4_ + MS_5_ + MS_6_). Phlebotomines caught in a light trap were considered a single sample.

### Shannon diversity index

To evaluate and compare differences in the composition of the sand fly community at each monitoring station, the Shannon‐Wiener diversity index (H') (Shannon, 1948) was used, and to assess whether there were significant differences between the diversity indices, the t‐test was applied, with a significance level of 5%, using the Past 3.16 software (Hammer et al., 2001).

### Analysis of collected sand fly species in the EPA and TFBR

Thematic maps were drawn to show the spatial distribution of the potential vectors collected in the study areas. The data were obtained by georeferencing the sampling sites using geographical coordinates obtained in the field with a portable Global Positioning System (GPS) device. Cartographic bases were obtained from the IBGE geographic database [https://downloads.ibge.gov.br/], and when available, regional and local bases were obtained directly from the Nova Iguaçu Municipal Department of the Environment, Agriculture, Economic Development, and Tourism.

All analyses were conducted using R version 4.4.2 (R Core Team, 2024). To assess spatial autocorrelation, global Moran's I was computed for each species, using a k‐nearest neighbours approach with k = 5 to ensure full connectivity of the spatial weight matrix given the limited number of sites. Moran's I and associated *p*‐values were calculated with the spdep package version 1.3–10 (Bivand & Wong, 2018). To map sites with higher abundance of each species, interpolated surfaces were generated for each species based on Inverse Distance Weighting (IDW). A regular spatial grid with 100 × 100 cells was created, buffered by approximately 500 metres from the sampling sites to aid visualization. IDW interpolation was performed separately for each species using the gstat package version 2.1–3 (Gräler et al., 2016). Final maps were designed in QGIS version 3.34.12‐Prizren (Graser et al., 2025) using standard WGS84 datum. SISA values were mapped with a white‐to‐dark red colour ramp from 0 to 1 to highlight subtle differences in lower abundance. Values were categorized as low (0.01–0.24), medium (0.25–0.74) or high (0.75–1).

### Legal terms

The research was authorized by the Biodiversity Authorization and Information System (SISBIO) under process no. 71038–1 (ICMBIO) and by the Department of Environment, Agriculture, Economic Development, and Tourism of the Municipality of Nova Iguaçu (SEMADETUR/CAPB) under process no. 2019/160731.

The owners signed an authorization form to collect the traps in their homes.

The data that support the findings of this study are openly available in Zenodo (Santana et al., 2025).

## RESULTS

Were collected during the period of the 7‐month, 2162 specimens identified, 50.8% of which were females. Sixteen species belonged to the genera *Psychodopygus* (3), *Nyssomyia* (1), *Psathyromyia* (3), *Evandromyia* (2), *Pintomyia* (2), *Micropygomyia* (1), *Migonemyia* (1), and *Brumptomyia* (3). The genus *Migonemyia* did not occur in the EPA, whereas the genus *Nyssomyia* was not detected in the TFBR.

*Psychodopygus hirsutus hirsutus* was the species with the highest SISA (0.41), followed by *Ps. davisi* with a value of 0.37. The possible vectors *Ps. hirsutus hirsutus, Ps. davisi, Ny. intermedia, Ps. ayrozai, Pi. fischeri, Ev. edwardsi* and *Mg. migonei* were found in different MSs with SISA values ranging from 0.02–0.41. These species accounted for 96% of the total number of specimens collected from the two study areas (Table 2).

**TABLE 2:** Total number, percentage and standardized index of species abundance of phlebotomines collected in Environmental Protection Area (EPA) and Tinguá Federal Biological Reserve (TFBR) in areas, Nova Iguaçu Municipality, Rio de Janeiro State, Brazil, from September 2019 to March 2020.

Species	Male	Female	Total	%	SISA
*Psychodopygus hirsutus hirsutus* ^a^	388	462	850	39.3	0.41
*Psychodopygus davisi* ^a^	417	520	937	43.3	0.37
*Nyssomyia intermedia* ^a^	154	37	191	8.8	0.18
*Psychodopygus ayrozai* ^a^	46	34	80	3.7	0.11
*Psathyromyia pascalei*	23	2	25	1.2	0.11
*Psathyromyia pelloni*	22	15	37	1.7	0.10
*Evandromyia edwardsi* ^a^	1	8	9	0.4	0.06
*Brumptomyia nitzulescui*	3	2	5	0.2	0.04
*Evandromyia termitophila*	1	3	4	0.2	0.03
*Pintomyia fischeri* ^a^	—	2	2	0.1	0.03
*Micropygomyia quinquefer*	—	4	4	0.2	0.03
*Migonemyia migonei* ^a^	2	4	6	0.3	0.02
*Brumptomyia brumpti*	—	2	2	0.1	0.02
*Psathyromyia lanei*	5	—	5	0.2	0.01
*Pintomyia misionensis*	—	4	4	0.2	0.01
*Brumptomyia cardosoi*	1	—	1	0.1	0.01
Total	1063	1099	2162	100	—

^a^ Possible vectors.

When analysing the collects in the different MSs, 75% (1622 specimens) of the specimens occurred in the EPA, a place with the highest number of collected specimens, and 25% (540 specimens) in the TFBR. The ratio between the total number of specimens and the number of collects was 33.8 specimens in the EPA and 30 in the TFBR.

Of the six MSs, only collections made at MS_1_ were negative, located in a peri‐urban area; at the other stations, 50.7%, 24.3%, 16%, 4.9% and 4.1% corresponded to the total number of specimens caught at MS_2_, MS_3_, MS_4_, MS_5_ and MS_6_, respectively.

**FIGURE 2:**
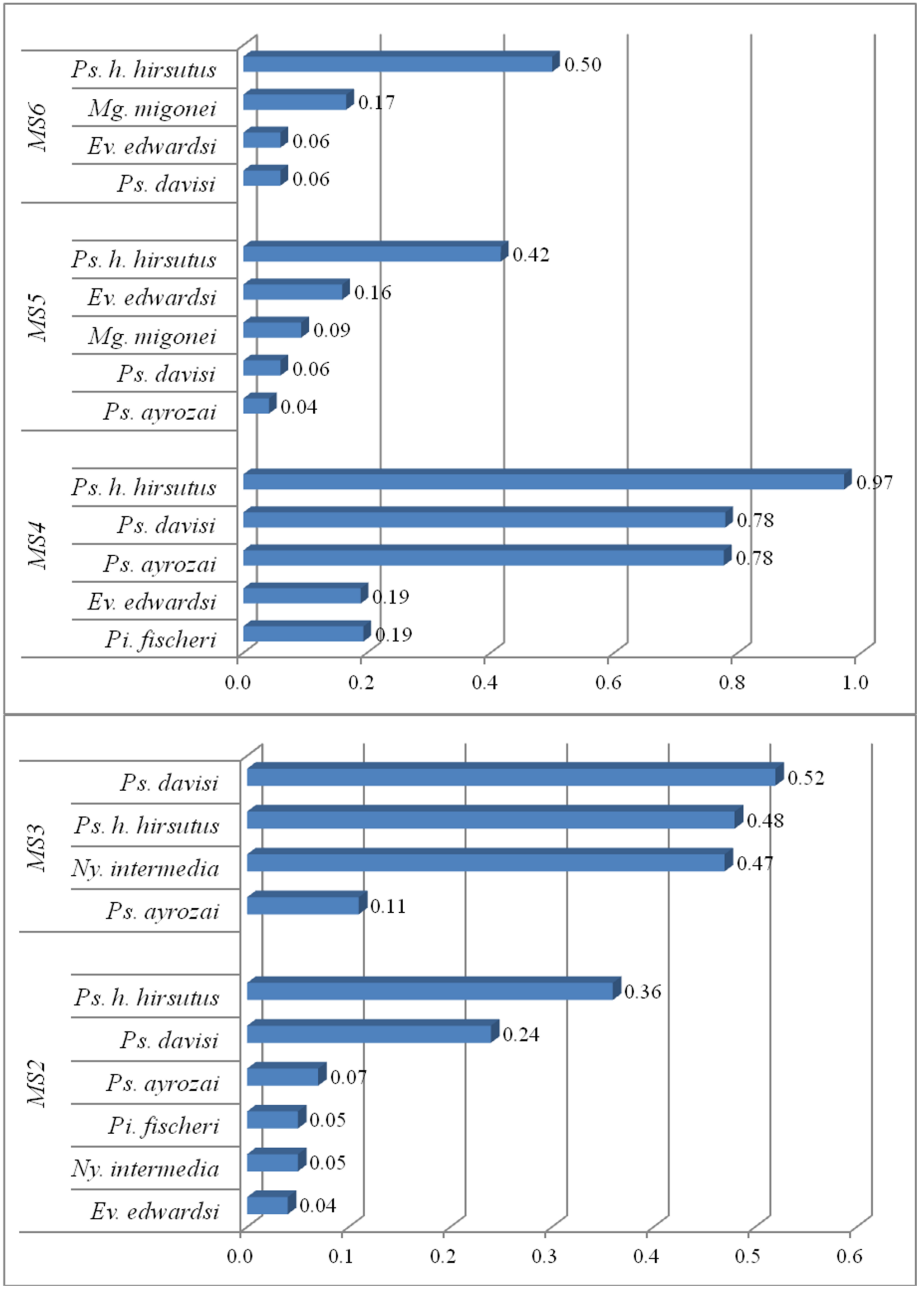
Standardized Abundance Index of potential (SISA) vector species collected in the Tinguá Environmental Protection Area (MS_2_ and MS_3_) and Tinguá Biological Reserve (MS_4_, MS_5_ and MS_6_), Nova Iguaçu Municipality, Rio de Janeiro State. Period from September 2019 to March 2020.

Of the total number of specimens collected in the EPA, 50.7% were at MS_2_, where *Ps. hirsutus hirsutus* (0.36) exhibited the highest SISA, followed by *Ps. davisi* (0.24). *Ps. ayrozai*, *Ny. intermedia*, *Pi. fischeri* and *Ev. edwardsi* also occurred at this station, with SISA values varying from 0.04 to 0.07. At MS_3_, where 24.3% of all specimens were collected, the SISA values of *Ps. davisi* were highest (0.52), followed by *Ps. hirsutus hirsutus* (0.48) and *Ny. intermedia* (0.47), respectively. *Ps. ayrozai* (0.11) was also detected at this station. In TFBR, specimen collections in MS4 (16%) were more efficient than in MS5 (4.9%) and MS6 (4.1%) In MS_4_, there were five potential vectors with their respective SISAs estimated at 0.97 (*Ps. hirsutus hirsutus*), 0.78 (*Ps. davisi* and *Ps. ayrozai*), and 0.19 (*Pi. fischeri* and *Ev. edwardsi*). In MS_5_, there were five vectors: *Ps. hirsutus hirsutus* (0.42), *Ev. edwardsi* (0.16), *Mi. migonei* (0.09), *Ps. davisi* (0.06), and *Ps. ayrozai* (0.04), whereas in MS_6_ there were *Ps. hirsutus hirsutus* (0.50), *Mi. migonei* (0.17), and *Ps. davisi* and *Ev. edwardsi* (0.06) (Figure 2).

**FIGURE 3:**
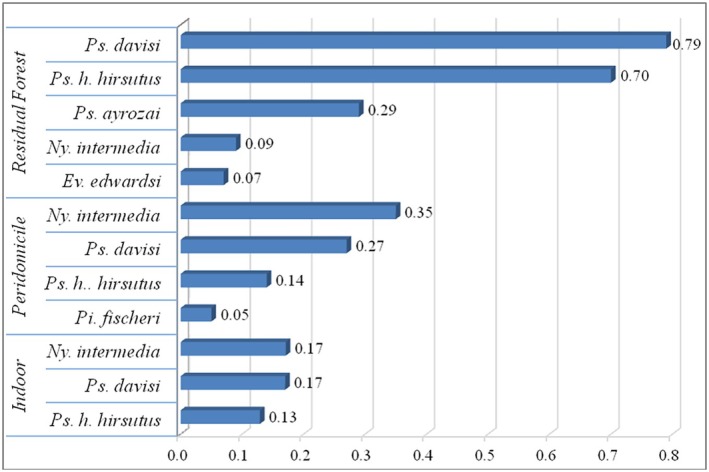
Standardized Abundance Index of potential vector species collected in the intradomicile, peridomicile and residual forest in the Environmental Preservation Area, Municipality of Nova Iguaçu, State of Rio de Janeiro. Period from September 2019 to March 2020.

When estimating the SISA indices of potential vectors at the three collect sites in the EPA, values of 0.17, 0.35 and 0.09 were observed for *Ny. intermedia* in the intradomicile, peridomicile, and residual forests, respectively; 0.13, 0.14 and 0.70 for *Ps. hirsutus hirsutus* in the intradomicile, peridomicile and residual forest, respectively; 0.17, 0.27 and 0.79 for *Ps. davisi* in the intradomicile, peridomicile, and residual forest, respectively; while *Pi. fischeri* indices, in the peridomicile (0.05) and residual forest were 0.29 for *Ps. ayrozai* and 0.07 for *Ev. edwardsi* (Figure 3).

**FIGURE 4:**
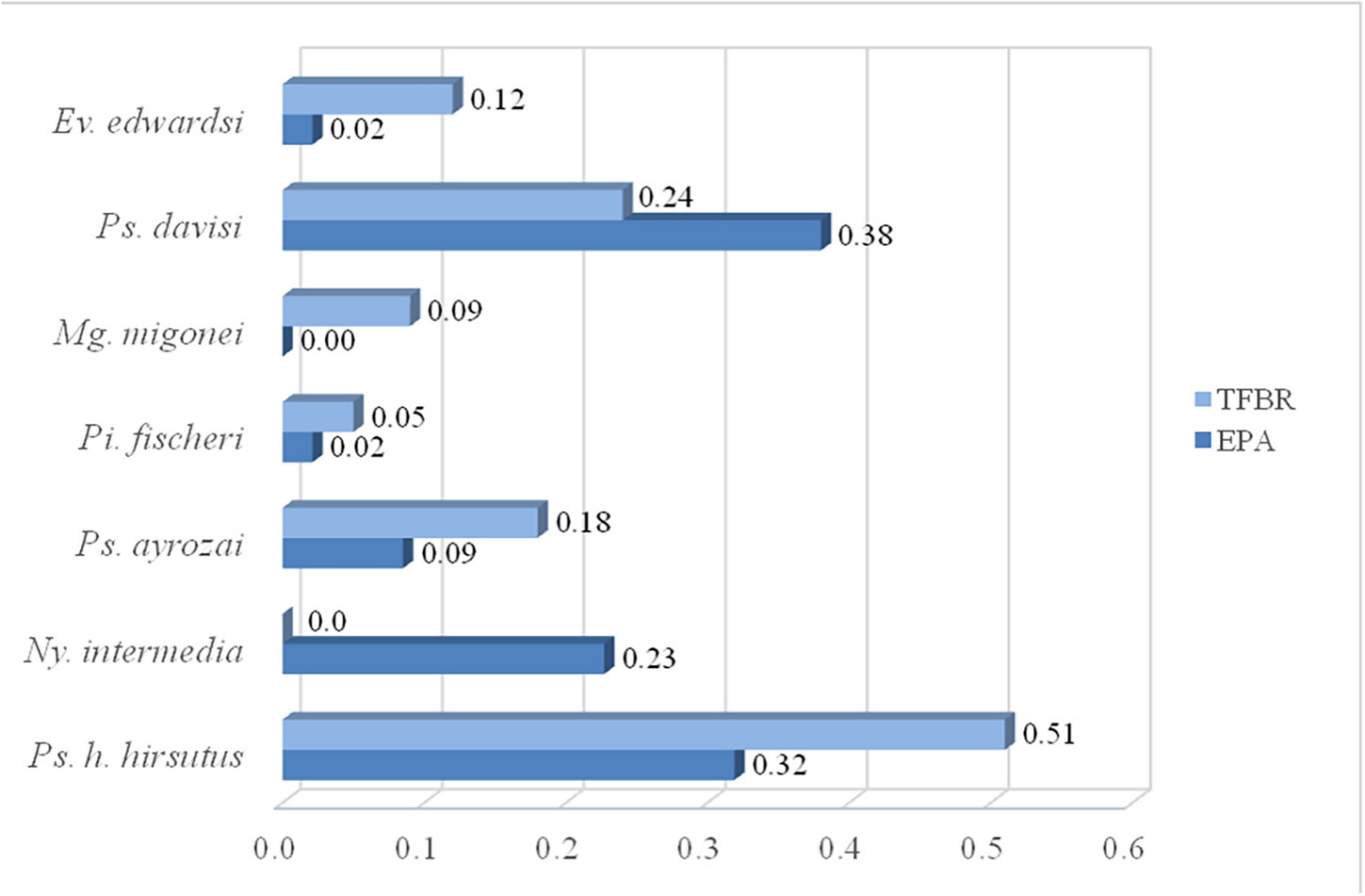
Standardized Abundance Index of Potential Vector Species Collected in the Tinguá Environmental Protection Area and Biological Reserve, Nova Iguaçu Municipality, Rio de Janeiro State. Period from September 2019 to March 2020.

*Psychodopygus davisi*, *Ps. hirsutus hirsutus, Ps. ayrozai, Pi. fischeri and Ev. edwardsi* occurred in the EPA and the TFBR. *Mg. migonei* did not occur in the EPA, and *Ny. intermedia* did not occur in the TFBR. The highest estimated SISA value in the EPA was 0.38 (*Ps. davisi*) followed by 0.32 (*Ps. hirsutus hirsutus*) and 0.23 (*Ny. intermedia*), while it was 0.51 (*Ps. hirsutus hirsutus*) and 0.24 (*Ps. davisi*) in the TFBR (Figure 4).

The area that presented the highest specific richness (number of species) was MS_5_ (TFBR), with S = 11, followed by the forest areas of MS_2_ (EPA) and MS_4_ (TFBR), both with S = 9, with the forest in MS_2_ standing out for representing the highest number of collected individuals (*N* = 1100). The greatest diversity was found in the peridomicile in MS_2_ (H' = 1.609), despite the lower number of collected individuals. Next, MS_5_ (tree hollow) stood out, the area with the greatest richness, with H' = 1.596; and MS_4_ (bamboo grove) with H' = 1.246. The area with the lowest specific richness was the intradomicile of MS_3_ (EPA) (S = 3), while the lowest diversity was found in MS_6_ (rocky slope/ TFBR) (H' = 0.431). This low diversity is mainly due to the dominance of *Ps*. *hirsutus hirsutus* in relation to the other species collected in the area. The lowest numbers of individuals collected were recorded in the peridomicile of MS_2_ and in the intradomicile of MS_3_, with *N* = 5 and *N* = 26, respectively.

The t‐test was used to assess whether there were significant differences between the diversities of the collect points. The results indicated that there was a significant difference between the following population diversities of the stations, according to the t‐test for H′ at 5% probability: the peridomicile and the forest of MS_2_; the peridomiciles of MS_2_ and MS_3_; the forests of MS_2_ and MS_3_, intradomicile of MS_3_, peridomicile of MS_3_ differs from MS_4_ and MS_5_; MS_4_ differs from MS_5_; and MS_6_ differs from all other stations.

**FIGURE 5:**
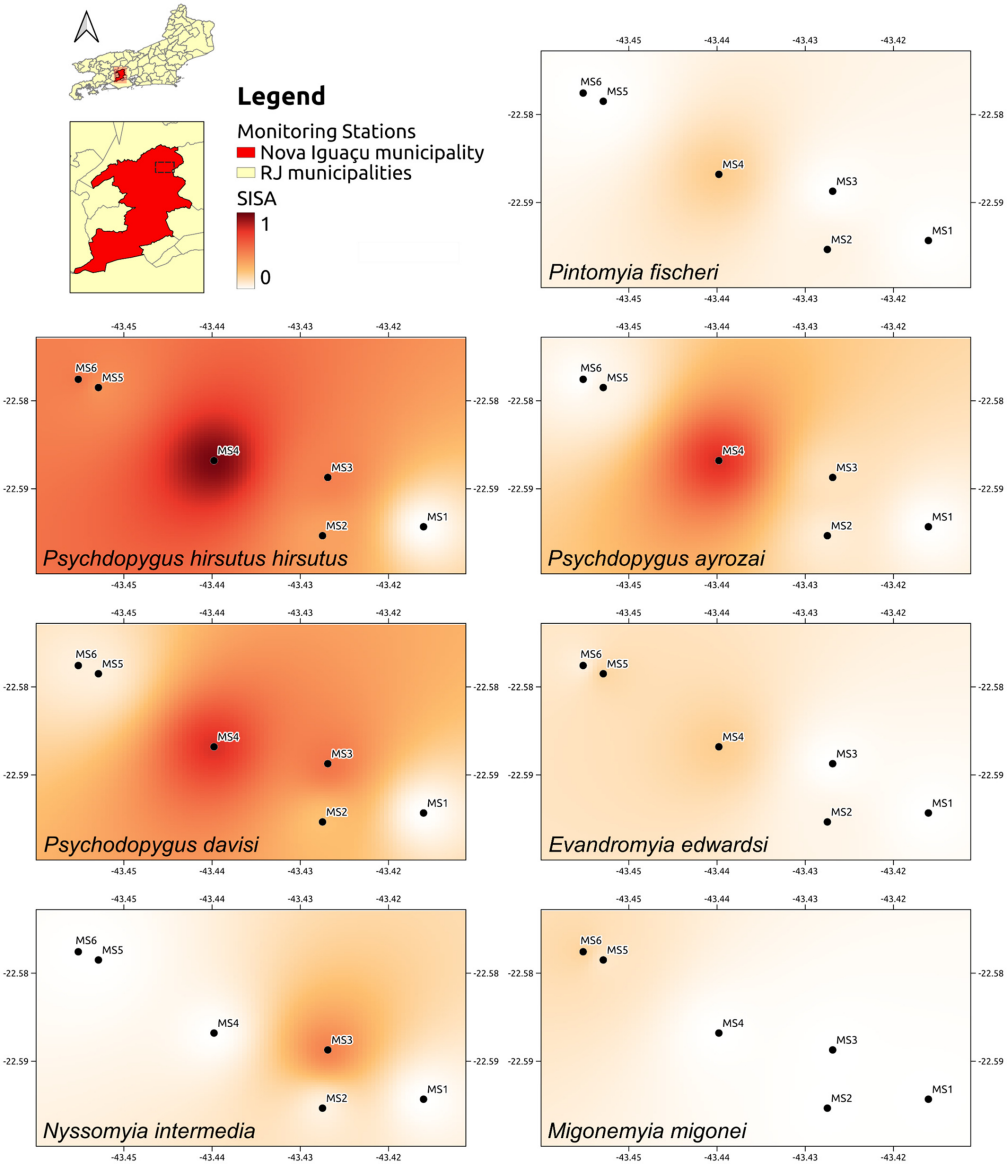
Interpolated abundance of the main vector species collected in the Municipality of Nova Iguaçu, State of Rio de Janeiro from September 2019 to March 2020.

The results of the Moran's I for all species were not statistically significant, indicating that the limited number of sampling sites was insufficient to assess spatial autocorrelation. Despite this, notable spatial patterns emerged in species abundance distributions: Among the seven studied species, *Ps*. *hirsutus hirsutus* was the most widely distributed and abundant, followed by *Ps*. *davisi* and *Ps*. *ayrozai*, with higher abundances at MS_4_, and medium to low SISA values at MS_2_, MS_3_, and MS_5_. In contrast, the remaining species *Ev*. *edwardsi*, *Mg*. *migonei*, *Ny*. *intermedia*, and *Pi*. *fischeri* were detected at low abundances across most sites, except for *Ny*. *intermedia*, which exhibited medium abundance at MS_3_. All species were absent from MS_1_ (Figure 5).

In total, 201 specimens from five MS_s_ were randomly selected and analysed for diagnosis of *Leishmania* spp. This included *Br. brumpti* (2) (MS_5_), *Ny. intermedia* (17) (MS_3_), *Ps. ayrozai* (6) (MS_2,3,4,5_), *Ps. davisi* (96) (MS_2,3,4,5,6_), *Ps. hirsutus hirsutus* (80) (MS_2,3,4,5,6_). Diagnosis of a single *Ps. hirsutus hirsutus* was positive for *L*. (*V*.) *braziliensis* in the peridomicile region (EPA, EM_3_), the positive sample was confirmed by hybridization with probes specific for *Leishmania* spp. Of this total, 25 females had visible blood residue: *Ny. intermedia* (1), *Ps. ayrozai* (3), *Ps. hirsutus hirsutus* (12), *Ps. davisi* (8) and *Br. brumpti* (1) were selected and analysed to determine the food sources. One specimen of *Ps. davisi* was diagnosed with the presence of blood from *Tamandua tetradactyla* (Tamanduá‐mirim) (GenBank access code: MW752259.1), collected in EM_5_ (TFBR). It is important to emphasize that a small sample of sandflies was analysed for both *the Leishmania* diagnosis and food sources.

## DISCUSSION

This study, conducted in the municipality of Nova Iguaçu, in the district of Tinguá, in the EPA and TFBR areas, was scheduled to take place over 12 months, although it was interrupted in March 2020 due to the COVID‐19 pandemic. Over the course of 7 months (September 2019 to March 2020), seven collects were made, and data were obtained based on 67 samples from different MSs.

Although the survey of phlebotomine fauna was conducted over a short period, the number of species in the TFBR (forest) was close to that observed in other studies conducted over longer periods in other reserves located in the State of Rio de Janeiro (Aguiar et al., 1996; Aguiar & Soucasaux, 1984).

There was little difference in the number of species found in the EPA and TFBR. However, in the latter, as it is a more preserved forest area, species diversity tends to be higher, according to studies carried out by Arias and Freitas (1982), Ready et al. (1986) and Azevedo et al. (2008).

*Psychodopygus hirsutus hirsutus* was the species with the highest abundance index and was present in both areas, with indices ranging from 0.36–0.48 in the EPA and 0.42 to 0.97 in the TFBR. The species occurred in the residual forest (EPA) and forest (TFBR), as well as in the intradomicile and peridomicile areas (EPA). In the peridomicile (MS_2_), one specimen had *L*. (*V*.) *braziliensis* DNA detected. *Psychodopygus hirsutus hirsutus* has wild habits, but is strongly attracted to humans, as demonstrated in studies carried out in the Serra dos Órgãos National Park (Aguiar & Soucasaux, 1984). Ryan et al. (1987) found, in the same species, in the Serra de Carajás “flagellates identified as subspecies *L*. (*V*.) *braziliensis* subspecies”. In a study conducted by Rangel et al. (1985) in Além Paraíba, a municipality in Minas Gerais close to the border with the State of Rio de Janeiro, there was only one specimen of *Ps. hirsutus hirsutus* infected with *Leishmania* of the subgenus *Viannia*.

*Psychodopygus davisi* was the second species with the highest abundance index and also occurred in both areas, with indices ranging from 0.24–0.54 in the EPA and 0.06 to 078 in the TFBR. The species were collected in the residual forest (EPA) and forest (TFBR), as well as in the intradomicile and peridomicile areas (EPA). One specimen was found feeding on *Tamandua tetradactyla* in the TFBR (MS_5_). It is important to highlight that *Tamandua tetradactyla* is a possible host and reservoir of *Leishmania* sp. and is also important for the maintenance of transmission cycles in wild and synanthropic environments (Roque & Jansen, 2014). Studies carried out by Rotureau (2006), Lainson et al. (1981), and Mimori et al. (1989) showed that *L*. (*V*.) *guyanensis* was isolated from this species of anteater in Brazil and *L*. (*Leishmania*) *amazonensis* in Ecuador. As early as the 1970s, flagellates have been found in the digestive tract of *Ps. davisi* (Shaw & Lainson, 1972). The importance of *Ps. davisi* as a potential vector was discussed by Gil et al. (2003) in an endemic area of the State of Rondônia and by de Souza et al. (2016) in the State of Pará, where the species was predominant, highly anthropophilic and found to be infected with *L*. (*V*.) *naiffi*.

*Nyssomyia intermedia* is considered the main vector of *L*. (*V*.) *braziliensis* in southeastern Brazil and is widely distributed in the State of Rio de Janeiro (Andrade Filho et al., 2007). In this study, the species occurred at all three collect sites in the EPA, which confirmed its adaptation to home and peridomiciliary environments where there are domestic animals, accumulation of organic matter, and forests altered by humans (Afonso et al., 2005; Araújo Filho et al., 1981; Carvalho et al., 2014; Forattini, 1953, 1973; Forattini et al., 1976; Gomes et al., 1989; Pirmez et al., 1997; Rangel et al., 1986). It is important to note that ATL is endemic to the municipality of Nova Iguaçu, with *Ny. intermedia* the main species found in the areas where this disease occurs (Santana, 2003). In the Municipality of Mesquita (formerly a district of the Municipality of Nova Iguaçu) and the State of Rio de Janeiro, Rangel et al. (1990) observed a predominance of *Ny. intermedia* and Afonso et al. (2005) found that this species is anthropophilic in peridomiciliary environments. Epidemiological studies of potential vectors in areas where ATL occurs in Municipalities of Rio de Janeiro have shown anthropogenic changes and a higher frequency of *Ny. intermedia* in hot and humid months, in addition to the fact that it has already been found to be naturally infected by *L*. (*V*.) *braziliensis*, where the species stands out as the main vector carrying out a household cycle and is generally followed by *Mg. migonei*, which acts as a secondary vector during the colder and drier months (Aguiar & Soucasaux, 1984; Aguiar & Vilela, 1987; Aragão, 1922, 1927; Azevedo et al., 2015; Rangel et al., 1990; Sabroza et al., 1975). The absence of *Ny*. *intermedia* in the forest environment may suggest that transmission by this sand fly species occurs in the domestic environment.

*Psychodopygus ayrozai* is an anthropophilic species found in the mountainous regions of southeastern Brazil and is more frequent during hot and humid months (Aguiar & Soucasaux, 1984). In the present study, *Ps. ayrozai* was found in the residual forest (MS_3_) and inside the reserves (MS_4_ and MS_5_) between December and February, leading to this species having the highest abundance index in MS_4_. This species has been identified as a vector for *L*. (*V*.) *naiffi* in the State of Pará (Lainson & Shaw, 1998; Rangel & Lainson, 2009), although it is not an anthropophilic phlebotomine species in the Amazon region (Lainson & Shaw, 1998; Rangel & Lainson, 2009). Specimens of this phlebotomine have also been found in *L*. (*V*.) *naiffi* in the States of Amapá and Rondonia (Arias et al., 1985; de Souza et al., 2016). In the Cerrado (state of Tocantins), the species was naturally infected with *L*. (*V*.) *braziliensis*, although the frequency was low and the authors suggested that *Ps. ayrozai* may play a secondary role in local epidemiology (Vilela et al., 2013).

*Pintomyia fischeri* occurs both in forest areas and in the domestic environment, where it occurs in domestic animal shelters (Aguiar et al., 1989; Moschin et al., 2013; Vieira et al., 2015, 2022). The species is identified as a secondary vector of *L*. *braziliensis* and suspected as *Leishmania* (*Leishmania*) *infantum* in the metropolitan region of São Paulo and in Porta Alegre, in Rio Grande do Sul (Galvis‐Ovallos et al., 2020; Rêgo et al., 2020). In the current study, this species occurred with a low abundance index in the chicken coop (MS_2_) and MS_4_ at 1578 m from MS_2_. Aguiar et al. (1989) and Vieira et al. (Vieira et al., 2015; Vieira et al., 2022) highlighted that, *Pi*. *fischeri* was the species with the greatest dispersion, as they observed, based on the ratio of collected females and males, that the species probably maintains its breeding grounds in the forest; however, they appear in the anthropic environment for hematophagy, while *Ny*. *intermedia* and *Mg*. *migonei* could be the species most adapted to the peridomiciliary environment. Vieira et al. (2022) in a study in the State of Rio de Janeiro showed the presence of the species at three collecting sites (home, peridomicile, and forest). However, the distance between the forest and the anthropogenic area, around 300 m, suggested that it was the species with the greatest dispersal and that it could be participating as a maintainer of the wild and home cycles, due to the small number of males collected in the home and peridomicile and the high number of males in the forest. Lainson (1983) suggested that this species could be an example of a phlebotomine that has adapted to environmental changes caused by human activity, maintaining the ability to transmit *L*. (*V*.) *braziliensis* among wild animals in the remnants of secondary forests that are still preserved. Barretto (1943) recorded the occurrence of this species in 41 municipalities in the State of São Paulo and hypothesized that it adapted from the wild to the home environment.

*Evandromyia edwardsi* is associated with cave environments, rock formations, forests, and wild animal shelters (Capucci et al., 2023; Castellon et al., 1995). The species had a positivity rate of 7.1% in the research carried out by Capucci et al. (2023) with the application of the PCR technique directed at the ITS1 target to detect *Leishmania* DNA. Sucen (2005) and Serra e Meira et al. (2022), found the species infected with *L*. *braziliensis* and also mentioned the presence of DNA from *L*. *amazonensis* and *L*. *infantum*. No caves were observed close to the MSs in our study area; however, the region has several rock formations and a large number of wild animal burrows. These may be the factors determining the occurrence of *Ev. edwardsi*, even with a low abundance rate, or other species of sand flies with wild habitats.

*Migonemyia migonei* is a wild species, preferably found in areas with abundant vegetation, although it can occur in domestic animal shelters and less frequently in secondary forests and capoeiras (Araújo Filho et al., 1981; Rangel et al., 1986; Rangel et al., 2018). In the present study, this species was not observed in the peridomicile (animal shelter) or the residual forest of the EPA. In this study, the species occurred in MS_5_ and MS_6_ with low abundance levels at altitudes of 399 and 424 m, respectively. Aguiar et al. (1993) and Aguiar et al. (1996) in studies carried out in the municipality of Itaguaí (Serra do Mar), state of Rio de Janeiro, observed the predominance of *Mg. migonei* in drier areas without banana plantations at an altitude of 300 m. The occurrence of this species in domestic animal shelters was also not observed, perhaps because our collections were carried out during the hot and rainy seasons. *Migonemyia migonei* has been found naturally infected by flagellates in the State of São Paulo (Pessoa & Coutinho, 1941; Pessoa & Pestana, 1940) and also by *L*. (*V*.) *braziliensis* in the Municipality of Baturité, State of Ceará (Azevedo et al., 1990; de Queiroz et al., 1991) and in the State of Rio de Janeiro (Ilha Grande and Jacarepagua) (Carvalho et al., 2014; Gouveia et al., 2012; Pita‐Pereira et al., 2005; Rangel & Lainson, 2009). The species was also associated with American Visceral Leishmaniasis when studies used molecular techniques (PCR) to identify infection by *L*. (*L*.) *infantum chagasi* in the states of Pernambuco and Ceará (Carvalho et al., 2010; Rodrigues et al., 2016).

## CONCLUSION

Finally, it is important to highlight the presence in the region of primary vectors, potential vectors, *L*. (*V*.) *braziliensis* and a natural reservoir in the APA and REBIO, drawing attention to the probability of a transmission cycle that could be both wild and domestic for ATL. The studied area has very few studies in the literature and the data obtained are of great importance for referencing and guiding managers of entomological and epidemiological surveillance on the importance of reinforcing and intensifying measures for the prevention and control of the disease, aiming at the protection of the resident population and tourists who visit the region.

## AUTHOR CONTRIBUTIONS

**Antônio L. F. Santana:** Conceptualization; writing – original draft; investigation; methodology; data curation; writing – review and editing. **Alfredo C. R. Azevedo:** Conceptualization; writing – review and editing; formal analysis. **Margarete M. S. Afonso:** Conceptualization; writing – review and editing. **Bruno M. Carvalho:** Conceptualization; writing – review and editing; formal analysis. **Vanessa R. Vieira:** Writing – review and editing. **Simone M. Costa:** Conceptualization; writing – review and editing. **Júlia S. Silva:** Formal analysis; conceptualization. **Thais A. Pereira:** Conceptualization; writing – review and editing; methodology; formal analysis. **Daniela P. Pereira:** Conceptualization; methodology; writing – review and editing; formal analysis. **Maurício L. Vilela:** Project administration; supervision.

## CONFLICT OF INTEREST STATEMENT

The authors declare no conflicts of interest.

## Data Availability

The data that support the findings of this study are openly available in Zenodo [https://doi.org/10.5281/zenodo.17467420].
